# A Retrospective Study of First-Line Therapy Involving Immune Checkpoint Inhibitors in Patients With Poor Risk Metastatic Renal Cell Carcinoma

**DOI:** 10.3389/fonc.2022.874385

**Published:** 2022-04-28

**Authors:** Hyunji Jo, Joohyun Hong, Hongsik Kim, Hye Ryeon Kim, Ghee Young Kwon, Kyung A. Kang, Sung Yoon Park, Chan Kyo Kim, Byung Kwan Park, Jae Hoon Chung, Wan Song, Minyong Kang, Hyun Hwan Sung, Hwang Gyun Jeon, Byong Chang Jeong, Seong Il Seo, Seong Soo Jeon, Hyun Moo Lee, Se Hoon Park

**Affiliations:** ^1^Division of Hematology and Oncology, Department of Medicine, Samsung Medical Center, Sungkyunkwan University School of Medicine, Seoul, South Korea; ^2^Department of Pathology and Translational Genomics, Samsung Medical Center, Sungkyunkwan University School of Medicine, Seoul, South Korea; ^3^Department of Radiology and Center for Imaging Sciences, Samsung Medical Center, Sungkyunkwan University School of Medicine, Seoul, South Korea; ^4^Department of Urology, Samsung Medical Center, Sungkyunkwan University School of Medicine, Seoul, South Korea

**Keywords:** immunotherapy, immune checkpoint inhibitors, renal cell carcinoma, IMDC poor-risk, overall survival, sunitinib

## Abstract

**Purpose:**

Patients with International Metastatic RCC Database Consortium (IMDC) poor risk metastatic renal cell carcinoma (mRCC) rarely respond to first-line tyrosine kinase inhibitors (TKIs) including sunitinib, and carries a very poor prognosis. In recent years, combination therapy involving immune checkpoint inhibitors (ICIs) have demonstrated superior efficacy to sunitinib in poor risk disease.

**Materials and Methods:**

In a retrospective study using a cancer chemotherapy registry, 206 consecutive patients with mRCC in the first-line setting were identified between Oct 2019 and Dec 2020. Sixty-one patients had a poor risk mRCC, and were treated with TKI monotherapy (n=36), nivolumab plus ipilimumab (n=16), or pembrolizumab plus axitinib (n=9). Endpoints included overall survival (OS), progression-free survival (PFS), response rate (RR), and safety.

**Results:**

Patients’ median age was 61 years and the median number of risk factors was 3 (range, 3-5). During a median 23.0 months of follow-up, the median OS was 24.3 months with ICI-based combinations and 14.8 months with TKI monotherapy, and the median PFS periods were 9.3 months and 3.4 months, respectively. An objective response occurred in 60% of the patients receiving ICI-based combinations and in 19% of those receiving TKI monotherapy (P=0.001). In the multivariate regression model, number of IMDC risk factors and the ICI-based combination therapy were independent prognostic factors for PFS. All-causality grade 3 or 4 adverse events were 44% for ICI-based combinations and 50% for TKI monotherapy.

**Conclusions:**

Among patients with poor risk mRCC, first-line ICI-based therapy showed significantly longer OS and PFS, as well as a higher RR, than TKI monotherapy.

## Introduction

Over the past two decades, tyrosine kinase inhibitors (TKIs) targeting the vascular endothelial growth factor receptor (VEGFR), including sunitinib (1) and pazopanib (2), are standards-of-care for patients with clear cell metastatic renal cell carcinoma (mRCC). However, some patients who receive first-line TKIs do not achieve clinical response, and show a rapid progression (3). Patients with an intrinsic resistance to TKIs, or poor risk disease, are supposed to have a limited benefit from first-line sunitinib, and although temsirolimus was suggested as an option (4), those with poor risk mRCC had a grim prognosis (3). These patient subgroups probably differ both clinically and biologically, and the International Metastatic RCC Database Consortium (IMDC) derived a risk model in the era of TKIs from a large patient cohort (5), with 6 independent predictive factors of poor survival including a performance status, an interval from time of RCC diagnosis to systemic therapy, hemoglobin level, calcium, neutrophil and platelet counts.

First-line treatment for mRCC has expanded in recent years to include immune checkpoint inhibitors (ICIs) including nivolumab plus ipilimumab (6) and pembrolizumab plus axitinib (7). As a result, current guidelines recommend these ICI-based doublets in patients with mRCC considered intermediate or poor risk groups, whereas for all IDMC risk categories pembrolizumab plus axitinib has emerged as a preferred standard regimen (8). In Korea, nivolumab plus ipilimumab and pembrolizumab plus axitinib were approved for the first-line therapy in mRCC in 2018 and 2019, respectively. Since the ICI-based doublets were not fully reimbursed by the national health insurance system before Sep 2021, our patients received the regimens at the discretion of the treating medical oncologists based on clinical and/or economic judgment. In patients not eligible for ICIs, or who cannot afford to the drug cost, VEGFR TKIs including sunitinib or pazopanib were still offered to those with poor risk disease.

Considering the grim prognosis of poor risk mRCC patients, and in an effort to generate real-world data in Korean mRCC patients, we performed a retrospective study using a prospectively collected cancer chemotherapy registry. Because prospectively-designed, randomized controlled trials (RCTs) comparing these ICI-based doublets are lacking, retrospective, or real-world studies seem to be an important source of data to allow the choice of an optimal treatment, enhance patient counseling, and generate hypothesis for future studies.

## Methods

In the present single-center, retrospective study, we collected and reviewed follow-up patient data from our cancer registry. Written informed consent was given by all patients prior to receiving first-line systemic therapy for their mRCC, according to institutional guidelines. The study protocol was reviewed and approved by the Samsung Medical Center (SMC, Seoul, Korea) institutional review board (SMC IRB no. 2021-08-054). The criteria for case inclusion were as follows: (1) histologically confirmed diagnosis of clear cell carcinoma arising from kidney, (2) presence of metastatic disease, (3) no prior systemic therapy except for adjuvant treatments, (4) poor risk disease, and (5) availability of clinical data at the time of beginning therapy and follow-up. We excluded patients who were enrolled in clinical trials to ensure the choice of therapy was at the discretion of the treating doctors. All the data was prospectively recorded and only the survival data was updated at the time of analyses.

IDMC poor risk was defined according to the IMDC criteria (5): (1) Karnofsky performance status <80%, (2) less than 1 year from time of RCC diagnosis to systemic therapy, (3) anemia (hemoglobin level <lower limit of normal [LLN], 12 g/dL), (4) hypercalcemia (corrected calcium >upper limit of normal [ULN], 10.2 mg/dL), (5) neutrophilia (neutrophil count >ULN, 7.0x10^9^/L), and (6) thrombocytosis (platelet count >ULN, 400x10^9^/L). According to the number of risk factors, patients were categorized into favorable (0), intermediate (1 or 2 factors), and poor (3 or more factors) risk groups. All patients received first-line therapy involving TKI monotherapy (sunitinib or pazopanib), nivolumab plus ipilimumab, or pembrolizumab plus axitinib. Dosages and therapy schedules of each regimen were determined according to the approved guidelines. Therapy was continued until disease progression or lack of clinical benefit, withdrawal of consent, justifiable withdrawal at the investigator’s discretion, or toxicity. Toxicities were graded according the National Cancer Institute (NCI) criteria (CTCAE). The dosage of the subsequent cycles was adjusted according to the toxic effects that developed during the preceding cycle. After the first-line therapy had failed, second-line therapy was recommended to all the patients if their performance status was preserved. According to the guidelines and department policies, all tumor measurements were assessed after every 3 months of therapy, by using an abdominopelvic computed tomography (CT) scan and other tests that were used initially to stage the tumor. Tumor response was evaluated according to the Response Evaluation Criteria for Solid Tumors (RECIST).

The primary endpoint of the present study was overall survival (OS). Secondary endpoints included progression-free survival (PFS), response rates (RR), and safety. The starting of OS and PFS was the first day of therapy. PFS and OS were estimated according to the Kaplan-Meier method and the statistical significance of survival curves between groups was tested with a log-rank test. To examine the impact of clinical and treatment variables on the outcomes of therapy, multivariate Cox regression models were used with covariates including age (below vs. ≥ median), gender, previous nephrectomy, presence of other histologic subtypes than clear cell carcinoma, lactate dehydrogenase (LD), weight loss (>5%) before therapy, number of involved sites (one vs. ≥2), sites of metastases (liver, bone), baseline number of IMDC risk factors (3 vs. >3), and therapy regimens. The potential presence of interaction effects between baseline parameters was tested by defining product terms for the respective factors in a regression model. All P values were two-sided, with P < 0.05 indicating statistical significance. Analyses were performed using the R for Windows v2.11.1 software (R Core Team, Vienna, Austria; http://www.r-project.org).

## Results

**Figure 1:**
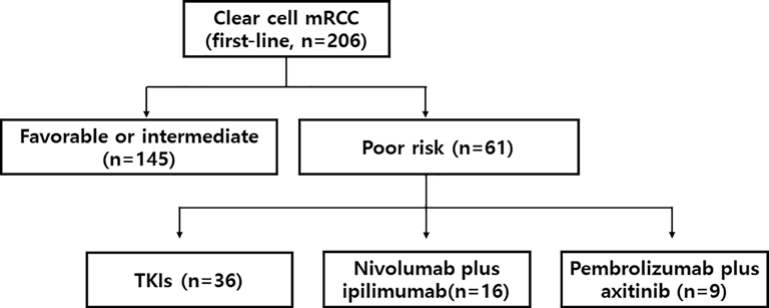
Study flow. mRCC denotes metastatic renal cell carcinoma. TKI denotes tyrosine kinase inhibitor.

We identified a total of 206 patients who were consecutively treated with first-line therapy for mRCC at the medical oncology department of SMC between Oct 2019 and Dec 2020. Among them, 61 patients were identified to have a poor risk mRCC (**Figure 1**). Fifty-nine percent (n=36) of patients received TKI monotherapy, and others (n=25) received ICI-based combinations (nivolumab plus ipilimumab, n=16; pembrolizumab plus axitinib, n=9). Baseline patient characteristics are listed in **Table 1**. The median number of IDMC risk factors was 3 (range, 3-5), and most commonly observed risk factors included anemia (84%) and the interval between diagnosis and therapy (71%). Forty-three (71%) patients had prior nephrectomy. Most common sites of metastases included lung and lymph nodes. At the time of analysis (Dec 2021), 57 (93%) patients had discontinued their first-line therapies.

**Table 1:** Patient characteristics of patients with poor risk, metastatic, clear cell renal cell carcinoma.

	All patients (n=61)	Tyrosine kinase inhibitors (n=36)	Checkpoint inhibitors (n=25)
Age, years			
Median (range)	63 (37-84)	66 (43-84)	58 (37-79)
Gender			
Male	47 (77%)	28 (78%)	19 (76%)
Female	14 (23%)	8 (22%)	6 (24%)
Prior nephrectomy	43 (71%)	30 (83%)	13 (52%)
Mixed histology	15 (25%)	8 (22%)	7 (28%)
Lactate dehydrogenase, IU/L			
Median (range)	284 (118-1,618)	284 (125-643)	283 (118-1,618)
Weight loss (>5%)	27 (44%)	17 (47%)	10 (40%)
No. of IMDC risk factors			
3	47 (77%)	28 (78%)	19 (76%)
4 or more	14 (23%)	8 (22%)	6 (24%)
IMDC risk factors			
Interval diagnosis/therapy <1y	43 (71%)	25 (69%)	18 (72%)
Karnofsky PS <80%	26 (43%)	14 (39%)	12 (48%)
Anemia	51 (84%)	30 (83%)	21 (84%)
Hypercalcemia	20 (33%)	11 (31%)	9 (36%)
Neutrophilia	18 (30%)	9 (25%)	9 (36%)
Thrombocytosis	32 (53%)	22 (61%)	10 (40%)
No. of metastatic sites			
1	27 (44%)	17 (47%)	10 (40%)
2 or more	34 (56%)	19 (53%)	15 (60%)
Metastatic sites			
Lymph nodes	22 (36%)	11 (31%)	11 (44%)
Lung	47 (77%)	26 (72%)	21 (84%)
Liver	8 (13%)	3 (8%)	5 (20%)
Bone	15 (25%)	9 (25%)	6 (24%)
Pancreas	9 (15%)	8 (22%)	1 (4%)
Brain	5 (8%)	2 (6%)	3 (12%)
Therapy regimen			
Sunitinib	29	29	
Pazopanib	7	7	
Nivolumab/ipilimumab	16		16
Pembrolizumab/axitinib	9		9

IMDC denotes the International Metastatic RCC Database Consortium. PS denotes performance status.

Patients received for a median of 3.8 months (95% CI, 3.1-4.5) of first-line therapy (**Table 2**). The most common reason for therapy discontinuation was progressive disease (75%). Overall, both TKIs and ICI-based combinations were generally well tolerated. Among 36 patients treated with TKI monotherapy, one patient discontinued therapy due to the development of acute myocardial infarction. In 25 ICI-treated patients, 4 patients discontinued therapy due to toxicities: grade 3 polyneuropathy (n=1), grade 4 hepatitis (n=1), grade 4 pneumonitis (n=1), and sudden death (n=1). A 58-year-old male patient was found dead at home in the midst of 7th cycle of pembrolizumab plus axitinib, with no clinical evidence of progression or adverse events demonstrated.

**Table 2:** Therapy compliance and safety.

	All patients (n=61)	Tyrosine kinase inhibitors (n=36)	Checkpoint inhibitors (n=25)
Therapy duration, mo			
Median	3.8	3.1	7.4
95% confidence interval	3.1-4.5	2.7-3.5	3.9-11.0
Reasons for discontinuation			
Progressive disease	46 (75%)	31 (86%)	15 (60%)
Toxicity	5 (8%)	1 (3%)	4 (16%)
Withdrawal	1 (2%)	1 (3%)	0
Physician recommendation	5 (8%)	2 (6%)	3 (12%)
Ongoing	4 (7%)	1 (3%)	3 (12%)
Overall grade 3 or 4 toxicity	29 (48%)	18 (50%)	11 (44%)
Corticosteroids use	7 (12%)	2 (6%)	5 (20%)
Treatment-related deaths	1 (2%)	0	1 (4%)

Among 61 patients with poor risk mRCC, 2 patients could not be evaluated for clinical responses because of early discontinuation of therapy. Objective responses to first-line therapy were noted in 29 patients (RR, 48%; 95% CI, 35-60%), including 4 complete responses seen in patients with ICI-based combination therapies. Patients who received TKI monotherapy were significantly less likely to respond to therapy (19% vs. 60%; P=0.001) compared to those who were treated with ICI-based combinations. RR was not significantly influenced by age, gender, weight loss, IMDC risk, or metastatic sites.

**Figure 2:**
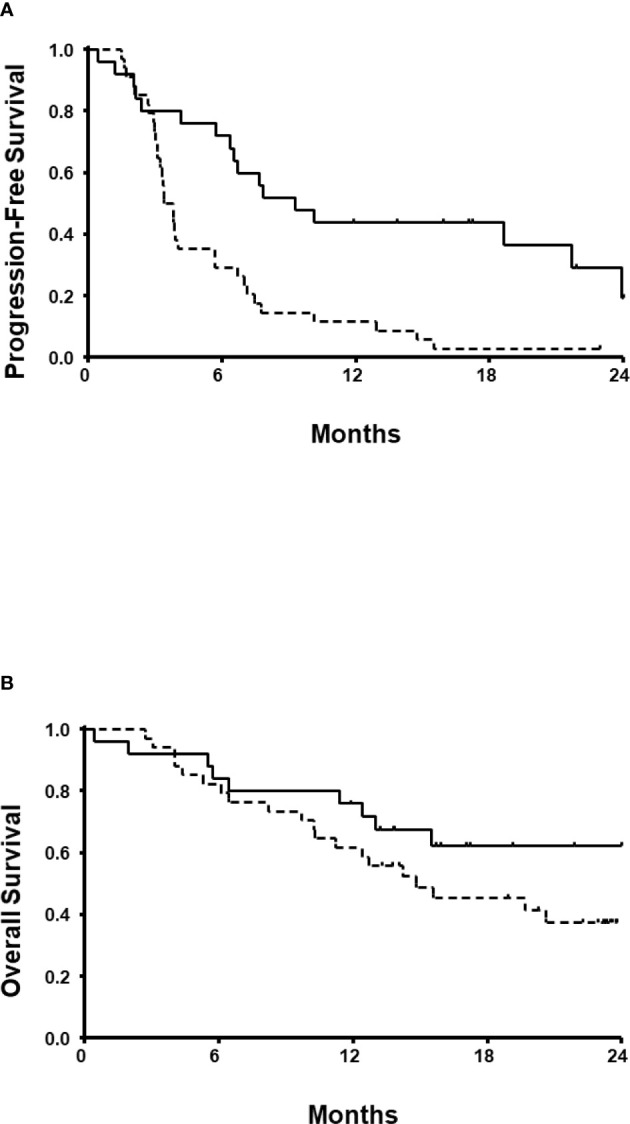
Progression-free survival **(A)** and overall survival **(B)**. Solid lines denote patients who received immune checkpoint inhibitors. Dotted lines denote patients who received tyrosine kinase inhibitor monotherapy.

With a follow-up duration of 23.0 months (95% CI, 22.1-24.4), the estimated median PFS and OS were 5.7 months (95% CI, 2.8-8.5) and 19.7 months (95% CI, 13.0-26.4), respectively. Both PFS (9.3 vs. 3.4 months; **Figure 2A**) and OS (24.3 vs. 14.8 months; **Figure 2B**) were longer in patients receiving ICIs than those receiving TKI monotherapy. In the univariate model, the estimated PFS was significantly longer for patients who received ICI-based combinations (P=0.001), and who had 3 risk factors (P=0.022). OS also was longer for patients who had 3 risk factors (P=0.042). However, no statistically significant difference in the OS was observed between ICI combinations and TKI monotherapy (P=0.162). A subsequent multivariate regression model revealed that independent prognostic factors for PFS were number of IMDC risk factors and the ICI-based combination therapy (**Table 3**). The presence of >3 IMDC risk factors was the only poor prognostic factor for OS.

**Table 3:** Multivariate analyses according to baseline clinical factors and therapy.

	Progression-free survival	Overall survival
No. of risk factors=3 vs. >3	HR 0.447 95% CI 0.220-0.906 P=0.026	HR 0.441 95% CI 0.196-0.992 P=0.048
Checkpoint inhibitors vs. TKIs	HR 0.339 95% CI 0.182-0.630 P=0.001	HR 0.567 95% CI 0.258-1.246 P=0.158

TKI denotes tyrosine kinase inhibitors.

For exploratory purposes, we compared PFS and OS in 25 patients treated with ICI-based combinations according to regimens given. No statistically significant differences in the median PFS (9.0 and 9.4 months, respectively) and OS (21.9 and 25.1 months, respectively) were observed between patients who received nivolumab plus ipilimumab and pembrolizumab plus axitinib. After first-line failure, second-line therapy was given to more than half of patients (n=34). Specifically, most patients received second-line TKIs (sunitinib, n=12; cabozantinib, n=8; axitinib, n=4), and novel therapeutics were given in 10 patients in the context of clinical trials. OS was longer in patients able to receive second-line therapy (25.3 vs. 12.9 months) than those without further therapy.

## Discussion

The main purpose of the present retrospective study was to investigate the real-world outcomes of patients with poor risk mRCC treated with different first-line therapy regimens. After regulatory approvals of first-line ICI-based therapy, significant prolongations of both PFS (9.3 vs. 3.4 months) and OS (24.3 vs. 14.8 months) were observed when compared to TKI monotherapy. This is consistent with the findings of the published trials of ICI-based first-line therapy (6, 7). Although interpretation of the present findings are limited by its retrospective nature and small sample size, the results provide a piece of evidence that patients with a poor-risk mRCC may derive an indisputable benefit from ICI-based combinations. Although it would be difficult to choose best first-line regimen from the present study or others, nivolumab plus ipilimumab and pembrolizumab plus axitinib provided similar outcomes.

Despite recent advances in the treatment of patients with clear cell mRCC, the prognosis of the IDMC poor risk patients remains challenging. Although current guidelines recommend first-line treatment with ICI in combination with TKI or nivolumab plus ipilimumab in this patient population (8), there remains controversy surrounding the choice of therapy regimens for poor risk disease. There is no head-to-head trial comparing the efficacy of the therapy options available including ICIs, TKIs, or a combination of both. Furthermore, in the IMDC retrospective study, there were no significant differences in first-line outcomes between nivolumab plus ipilimumab and ICI plus VEGFR TKIs (9). ICI plus TKI may be preferred in patients with highly symptomatic disease and a rapid clinical response is required, which may be offered by the TKI component of the regimen. One may consider a durable treatment response to be important as there is long-term follow-up data to demonstrate the durable response and survival benefit with nivolumab plus ipilimumab (6). Toxicity is also an important consideration given the balance between higher rates of immune-related adverse events associated with ICIs and the possibility of symptomatic deteriorations with TKIs.

In addition to clinical factors, appropriate patient selection based on molecular markers is one of the most extensively studied areas in clinical research. While PD-L1 expression is not considered a predictive marker as patients with PD-L1 negative tumors also benefit from ICI therapy, and the heterogeneity in PD-L1 testing methods adds complexity to this issue. Extensive work is ongoing to identify possible molecular markers, including the tumor mutation burden, immune infiltrates in the tumor microenvironment, or gene signatures, that could be related to sensitivity or resistance to ICIs (10), as well as specific genomic subtypes harbored in different risk groups (11).

More recently, more than a few novel combination therapy regimens have demonstrated improved survival outcomes (12–15), all of which compared the efficacy of ICI-based therapy with sunitinib as the control, which is no longer considered the standard of care in this patient population. As seen in these clinical trials involving therapeutic strategies, further advances in the treatment of poor risk mRCC will only be achieved with better patient selection. Emerging science and the knowledge of disease may further guide us to enhance individualized therapy for patients with mRCC.

## Data Availability Statement

The raw data supporting the conclusions of this article will be made available by the authors, without undue reservation.tf

## Author Contributions

All authors contributed conception and design of the study. GK, KK, SYP, CK, BP, JC, WS, MK, HS, HGJ, BJ, SS, SJ, HL, and SHP acquired the clinical data. HJ and SHP conducted the statistical analysis. HJ, JH, HK, HRK, and SHP analyzed and interpreted the data. HJ and SHP drafted the manuscript. All authors read and approved the submitted version.

## Funding

This word was supported by Samsung Medical Center (Seoul, Korea) Research Fund (OTA1602441, OTA1702441).

## Conflict of Interest

The authors declare that the research was conducted in the absence of any commercial or financial relationships that could be construed as a potential conflict of interest.

## Publisher’s Note

All claims expressed in this article are solely those of the authors and do not necessarily represent those of their affiliated organizations, or those of the publisher, the editors and the reviewers. Any product that may be evaluated in this article, or claim that may be made by its manufacturer, is not guaranteed or endorsed by the publisher.
